# O.1.3-3 Factors related to the implementation and scale-up of physical activity interventions in Ireland: a qualitative study with policy makers, funders, researchers and practitioners

**DOI:** 10.1093/eurpub/ckad133.100

**Published:** 2023-09-11

**Authors:** Joey Murphy, Fiona Mansergh, Gráinne O'Donoghue, Femke van Nassau, Jemima Cooper, Caera Grady, Niamh Murphy, Enrique Garcia Bengoechea, Marie Murphy, Benny Cullen, Catherine Woods

**Affiliations:** Centre for Exercise, Nutrition and Health Sciences, School for Policy Studies, University of Bristol, UK; Department of Health, Healthy Ireland, Ireland; School of Public Health, Physiotherapy & Sports Science, University College Dublin, Ireland; Department of Public and Occupational Health, Amsterdam Public Health research Institute, Amsterdam UMC, Vrije Universiteit Amsterdam, The Netherlands; Department for Health, University of Bath, UK; Physical Activity for Health Research Cluster, Department of Physical Education and Sport Sciences, University of Limerick, Ireland; Department of Sport and Exercise Science, South East Technological University, Ireland; Physical Activity for Health Research Cluster, Department of Physical Education and Sport Sciences, University of Limerick, Ireland; Research & Innovation Unit, Sport Ireland, Ireland; Centre for Exercise Medicine, Physical Activity and Health, Sports and Exercise Sciences Research Institute, Ulster University, Jordanstown Campus, UK; The Physical Activity for Health Research Centre, University of Edinburgh, UK; joey.murphy@bristol.ac.uk; Research & Innovation Unit, Sport Ireland, Ireland; Physical Activity for Health Research Cluster, Department of Physical Education and Sport Sciences, University of Limerick, Ireland

## Abstract

**Purpose:**

Current literature reports a gap between development of effective interventions to promote physical activity and their systematic uptake into real-world settings. Factors relating to implementation and scale-up of physical activity interventions have been examined, however the perspectives of multiple stakeholders from different domains are not well researched. The purpose of this study was to examine the perceived factors related to physical activity intervention implementation and scale-up in different domains from different stakeholders on the island of Ireland.

**Methods:**

Intervention providers, coordinators, researchers, funders and policy makers in Ireland were invited to take part in a semi-structured interview exploring factors related to the implementation and scale-up of eleven different physical activity interventions. A thematic analysis was conducted to identify factors related to the implementation and scale-up of the included interventions. The data collection and analysis were guided by the Consolidated Framework for Implementation Research.

**Results:**

Thirty-eight participants took part in the interviews (11 providers, 11 coordinators, 6 researchers, 6 policy makers and 4 funders) exploring factors related to physical activity intervention implementation and scale-up. Themes identified related to intervention 1) planning (e.g. intervention development and practical considerations); 2) delivery (e.g. organisational structures, staffing and delivery resources); 3) reflection, evaluation and updating of the intervention; and 4) scale-up (e.g. ensuring fidelity and scale-up considerations). Furthermore, participants referred to the ongoing commitment, engagement, and support needed from relevant stakeholders, communities and researchers throughout the implementation process.

**Conclusions:**

This research highlights multiple factors, that are inter-related, and influence the implementation of PA interventions, but also identifies many strategies that can be utilised to enable greater likelihood of success. Future research and practice needs to consider how different factors are experienced at different implementation stages and by the different stakeholder groups involved. As one participant noted, “The intervention doesn’t go into an ideal world, it goes out into a pragmatic world”, highlighting the need to understand what implementation strategies work for who and in what contexts.

**Funding Source:**

This work was funded by the Health Research Board (HRB APA-2017–030).

